# Impacts of prenatal nutrition on animal production and performance: a focus on growth and metabolic and endocrine function in sheep

**DOI:** 10.1186/s40104-017-0205-1

**Published:** 2017-09-01

**Authors:** Prabhat Khanal, Mette Olaf Nielsen

**Affiliations:** 1 Department of Veterinary and Animal Sciences, Faculty of Health and Medical Sciences, University of Copenhagen, Grønnegårdsvej 3, 1st floor, DK-1870 Frederiksberg C, Denmark; 2 Current address: Department of Nutrition, Faculty of Medicine, Transgenic Animal and Lipid Storage, Norwegian Transgenic Centre (NTS), University of Oslo, Oslo, Norway

**Keywords:** Adipose tissue, Endocrine function, Foetal programming, Metabolic function, Sheep

## Abstract

The concept of foetal programming (FP) originated from human epidemiological studies, where foetal life nutrition was linked to health and disease status later in life. Since the proposal of this phenomenon, it has been evaluated in various animal models to gain further insights into the mechanisms underlying the foetal origins of health and disease in humans. In FP research, the sheep has been quite extensively used as a model for humans. In this paper we will review findings mainly from our Copenhagen sheep model, on the implications of late gestation malnutrition for growth, development, and metabolic and endocrine functions later in life, and discuss how these implications may depend on the diet fed to the animal in early postnatal life. Our results have indicated that negative implications of foetal malnutrition, both as a result of overnutrition and, particularly, late gestation undernutrition, can impair a wide range of endocrine functions regulating growth and presumably also reproductive traits. These implications are not readily observable early in postnatal life, but are increasingly manifested as the animal approaches adulthood. No intervention or cure is known that can reverse this programming in postnatal life. Our findings suggest that close to normal growth and slaughter results can be obtained at least until puberty in animals which have undergone adverse programming in foetal life, but manifestation of programming effects becomes increasingly evident in adult animals. Due to the risk of transfer of the adverse programming effects to future generations, it is therefore recommended that animals that are suspected to have undergone adverse FP are not used for reproduction. Unfortunately, no reliable biomarkers have as yet been identified that allow accurate identification of adversely programmed offspring at birth, except for very low or high birth weights, and, in pigs, characteristic changes in head shape (dolphin head). Future efforts should be therefore dedicated to identify reliable biomarkers and evaluate their effectiveness for alleviation/reversal of the adverse programming in postnatal life. Our sheep studies have shown that the adverse impacts of an extreme, high-fat diet in early postnatal life, but not prenatal undernutrition, can be largely reversed by dietary correction later in life. Thus, birth (at term) appears to be a critical set point for permanent programming in animals born precocial, such as sheep. Appropriate attention to the nutrition of the late pregnant dam should therefore be a priority in animal production systems.

## Background

The term ‘foetal metabolic programming’ was defined in the early 1990s as a phenomenon linking long-term adverse health consequences in animal species with adverse nutritional exposures in utero [1, 2]. In the past, foetal programming (FP) and its long-term impacts have been evaluated particularly from a human health and disease perspective [3, 4], and such studies have revealed that FP has implications for a wide range of body functions, which are also key determinants of animal productivity. However, knowledge about the potential long-term implications of FP for animal productivity is still scare. Such knowledge is needed in order to assign the best management strategies (postnatal feeding, culling, etc.) to minimize implications of adverse FP for animal productivity and avoid possible trans-generational transfer of undesirable FP outcomes. In this review we will primarily focus on what has been found in sheep, where the long-term implications of foetal life malnutrition for development and metabolic and endocrine functions later in life have been extensively studied. Furthermore, we will also evaluate to what extent the diet fed in postnatal life can influence the phenotypic manifestation of the prenatal FP. In this regards, observations from other species will only be included where appropriate.

## Animal experimental approaches to the study of foetal programming

In the past, early nutritional programming has mostly been investigated in rodent models with a focus on the long-term implications for health and disease risk in humans. However, FP is one of the rare areas of research where sheep has also been used quite extensively as a model for humans [5–10] due to the similarities in the foetal developmental trajectory and physiological maturity at birth. Pig is another farm animal commonly used as a model for humans studies regarding FP [11], but the pig is born less physiologically mature than humans and ruminant offspring. Less frequently, non-human primates [12] have been used.

**Fig. 1:**
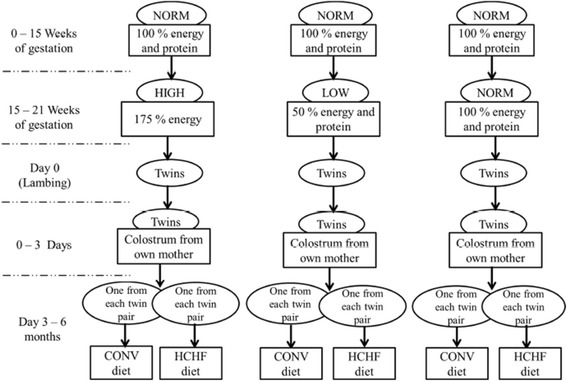
The experimental design of the Copenhagen sheep model showing different nutritional and dietary interventions during late gestation and early postnatal life in sheep (obtained from Khanal et al., 2014 [10]). Late gestational nutrition groups: HIGH, fulfilling 150% of Danish requirements for energy and 110% of requirements for protein; LOW, fulfilling only 50% of requirements for energy and protein; NORM; fulfilling 100% requirements for energy and protein. Early postnatal (from 3 d after birth until 6 mo of age) nutrition groups: one lamb from each twin pair was allocated to a HCHF diet (high-starch-high fat consisting of a milk replacer-dairy cream mix supplemented with rolled maize), and the other was fed a CONV (conventional/moderate, hay-based diet; growth rate of appr. 225 g/d) diet

We developed the Copenhagen sheep model [9, 10] to be able to study the long-term impacts of foetal over- and undernutrition in late gestation, and further to study how postnatal manifestations of FP are affected by the diet received in early postnatal life. In the studies based on this model, twin-pregnant ewes were exposed to adequate nutrition (NORM; 100% of Danish daily energy and protein requirements), undernutrition (LOW; 50% of NORM for energy and protein requirements) or overnutrition (HIGH; 150% of energy and 110% of protein requirements) during the last 6 wk of pregnancy (term ~147 d). When the twin lambs were born, they received colostrum within 3 h of birth, and suckled their dams at will for the first 3 days after parturition. Thereafter, the dam was removed and the lambs artificially reared until 6 mo of age (after puberty) on two different diets: one lamb from each twin pair received a moderate, conventional diet (CONV, consisting of good quality hay sufficient to achieve moderate growth rates of 225 g/d with a milk replacer until 8 wk of age), whereas the other lamb received an energy dense, high-starch-high-fat diet (HCHF, consisting of rolled maize and a dairy cream-milk replacer mix (1:1) fed ad libitum up to a maximum daily intake of 1 kg and 2.5 kg, respectively) (Fig. 1). From 6 mo of age until adulthood at 2–2.5 yr of age, all sheep were fed the same moderate grass/hay based diet. To minimize the potential paternal impacts in regards to foetal programming of maternal nutrition, rams used for mating the ewes prior to an exposure to prenatal nutritional treatments were of the same breed and similar ages and body weights and they were reared under similar management conditions. With this experimental design, it was possible to evaluate whether long-term adverse outcomes of foetal malnutrition and excessive fat deposition in early postnatal life can be reversed by nutritional intervention later in life. Details of the type of feeds used and the nutritional composition of the experimental diets are shown in Table 1.

**Table 1:** Different types of experimental feeds and their chemical composition and energy content

Feeds	DM, %	Ash, % of DM	aNDF, % of DM	ADF, % of DM	ADL, % of DM	CP, % of DM	Cfat, % of DM	DE, MJ/ kg DM
Sheep diet during late gestation								
Hay	91.4	5.6	47.7	27	3.1	20.8	4.8	13.7
Barley	89.0	2.3	14	6	1.1	12.5	3.1	17.1
Concentrate	87.7	7.7	25.8	18	2.8	15.3	3.8	12.8
Lamb diet during early postnatal life until puberty								
Hay	93.1	6.8	50.4	32.3	3.5	19.1	3.7	13.5
Maize	89.5	0.6	4.1	<5	0.9	8.5	1.9	16.3
Milk powder	95.6	7.1	-	-	-	22.5	23.6	19.2
Cream	42.9	0.8	-	-	-	4.3	38.0	30.5

These are the types of feeds used in diets for experimental animals in the Copenhagen sheep model; the table was obtained from Khanal et al., 2014 [10] with modifications. DM, dry matter; aNDF, amylase-treated neutral detergent fiber; ADF, acid detergent fiber; ADL, acid detergent lignin; CP, crude protein; Cfat, crude fat; DE, digestible energy

It should be noted that we characterized the long-term consequences of pre- and postnatal nutrition mismatch scenarios in a ruminant animal species without disrupting rumen fermentation or compromising animal health. Nutritional manipulations in our experiments were done during the late gestation period (third trimester), which is the period of extensive quantitative foetal growth [13] where many endocrine organs and tissues are matured, including adipose tissues [14]. Although nutritional insults during all stages of gestation can influence body functions of the offspring later in life [5, 15, 16], late gestation is the time window, where FP is most likely to occur in precocial farm animal species given birth to multiple offspring, such as sheep, due to the dramatic rise in nutrient requirements for the foetuses in late gestation [13, 17]. Although our studies were designed from a human health perspective, the results obtained allow us to evaluate how programming outcomes may affect both animal growth and metabolic and endocrine functions of importance for animal productivity, including the timing of manifestations. Such knowledge can help to refine nutritional strategies applied in livestock production [18].

## Impacts of maternal malnutrition on postnatal growth and organ and tissue development and function in growing animals

### Growth characteristics

Historically, birth weight has been used as a marker to identify individuals at risk of having undergone adverse FP [19]. Birth weight is in itself a poor indicator of nutritional programming, since it provides little information about body composition, adiposity and potentially altered body functions. Moreover, nutritional insults interfering with foetal growth during the earlier stages of gestation may not be reflected in changes in birth weight if catch-up growth occurs during later stages of gestation [6, 20]. However, it has been shown in several studies that undernutrition in late gestation can result in animals being born small for gestational age. In sheep, birth weights have been reduced in different experiments by 10–18% under controlled conditions, when dams were fed only 50–70% of their daily energy requirements during late gestation (see e.g. [9, 21]), and in goats kid birth weight was reduced by ~10% when dams were exposed to poor grazing conditions during the last 4 wk pre-partum [22]. Lower birth weight has in different studies been associated with reduced survival rate [23], poorer growth rate during the suckling period and 24% lower weaning weight at 14 wk of age in sheep [21]. These findings may partly be explained by poorer mammary development of dams malnourished in late gestation [24], leading to a reduction in colostrum and early lactation milk production [9, 25, 26]. However, in other studies in sheep where the dietary intake of offspring was controlled after birth (artificial rearing), the postnatal growth appeared to be entirely determined by the postnatal and not the prenatal level of nutrition [9, 10, 27, 28]. However, postnatal growth does appear to terminate earlier in individuals with a history of late gestation undernutrition, resulting in smaller adult body size [27]. In cattle, low birth weight (28.6 vs. 38.3 kg) or slow growth till weaning led to lowered body (56 or 46 kg less, respectively) and carcass weights (32 or 40 kg less, respectively) at slaughter at 30 mo of age [29]. Thus, proper attention should be given to ewe nutrition in the late gestational period to ensure not only optimal foetal growth, but also a desired level of colostrum and milk production. It may therefore be beneficial to consider supplementary milk feeding after birth in suckling individuals which were exposed to prenatal undernourishment to improve their postnatal immunization and growth performance.

### Skeletal muscle development and function

Proper growth of skeletal muscle and lean carcass mass in slaughter animals are important production traits for the livestock industry. Muscle fibre formation commences during the embryonic stage and, in animal species born precocial, the formation of secondary muscle fibers is concluded during mid-gestation [30]. Thus, in sheep, and other animals born precocial, conclusion of myotube formation and establishment of the final number of muscle fibres occurs prior to the onset of the third trimester [14, 31]. In other farm animal species, myogenesis may occur over a larger part of gestation, for example in pigs, where muscle fibre hyperplasia is not concluded until 95 d of the 114-d gestation period [32]. It must be anticipated, therefore, that foetal myofibre formation in such species may be affected by adverse nutritional insults during a greater part of the gestation. In ruminant animals, foetal undernutrition during the first part of gestation, when myogenesis takes place, has been shown to reduce the formation and number of secondary muscle fibres [15, 31]. In lambs exposed to undernutrition from 28 to 78 d of gestation, the reduced number of total secondary myofibers was reconizable at 8 mo of age [15], and in another study it was shown that undernutrition from 30 to 70 d of gestation altered muscle characteristics (fewer fast fibres and more slow fibres in the *longissimus* and *vastus lateralis* muscles) in new-born lambs [33]. In cows, improving the nutritional status of pregnant cows (improved pasture conditions) during mid to mid-late gestation (120–150 through 180–210 d of gestation; term ~280 d) improved carcass characteristics (tenderness) and also increased live and hot carcass weight in steers [34].

In contrast, exposure to malnutrition after myofibre formation has been completed does not appear to have major implications for muscle development and function postnatally. Thus, nutrient restriction (to 50% of daily requirements) between 85 and 115 d of gestation decreased muscle weight in lambs without affecting muscle fibre number [33]. Similarly, in adult sheep, we have not been able to demonstrate any changes in muscle mass or expression of markers for metabolic function in muscle of adult offspring that could be related to a history of late gestational foetal undernutrition (50% reduction in maternal energy and protein supply relative to recommendations) [35]. In conclusion, whether gestational malnutrition will have implications for myogenesis thus appears to depend on the timing of the nutritional insults relative to the conclusion of myotube formation in utero. The nutritional programming of skeletal muscle development prior to the conclusion of myofibre development appears to be permanent, whereas malnutrition in late gestation in precocial animal species does not have long-term consequences for skeletal muscle development.

### Adipose tissue deposition

In precocial animal species such as sheep, the major part of foetal adipogenesis and adipose tissue differentiation takes place during the last part of gestation [36–38]. Thus, if foetal nutrition should have implications for adipose tissue development, it would most likely be during the late gestation period. This has received much less attention in relation to FP in farm animals than muscle development, presumably due to the greater economic importance of the latter.

**Fig. 2:**
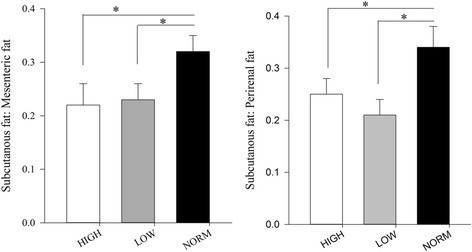
Impacts of late gestational over- and undernutrition on fat deposition patterns in adolescent (6 months old) offspring (left panel: subcutaneous to mesenteric fat ratio; right panel: subcutaneous to perirenal fat ratio) (obtained from Khanal et al. [10]). For HIGH, NORM and LOW, see legends for Fig. 1

**Fig. 3:**
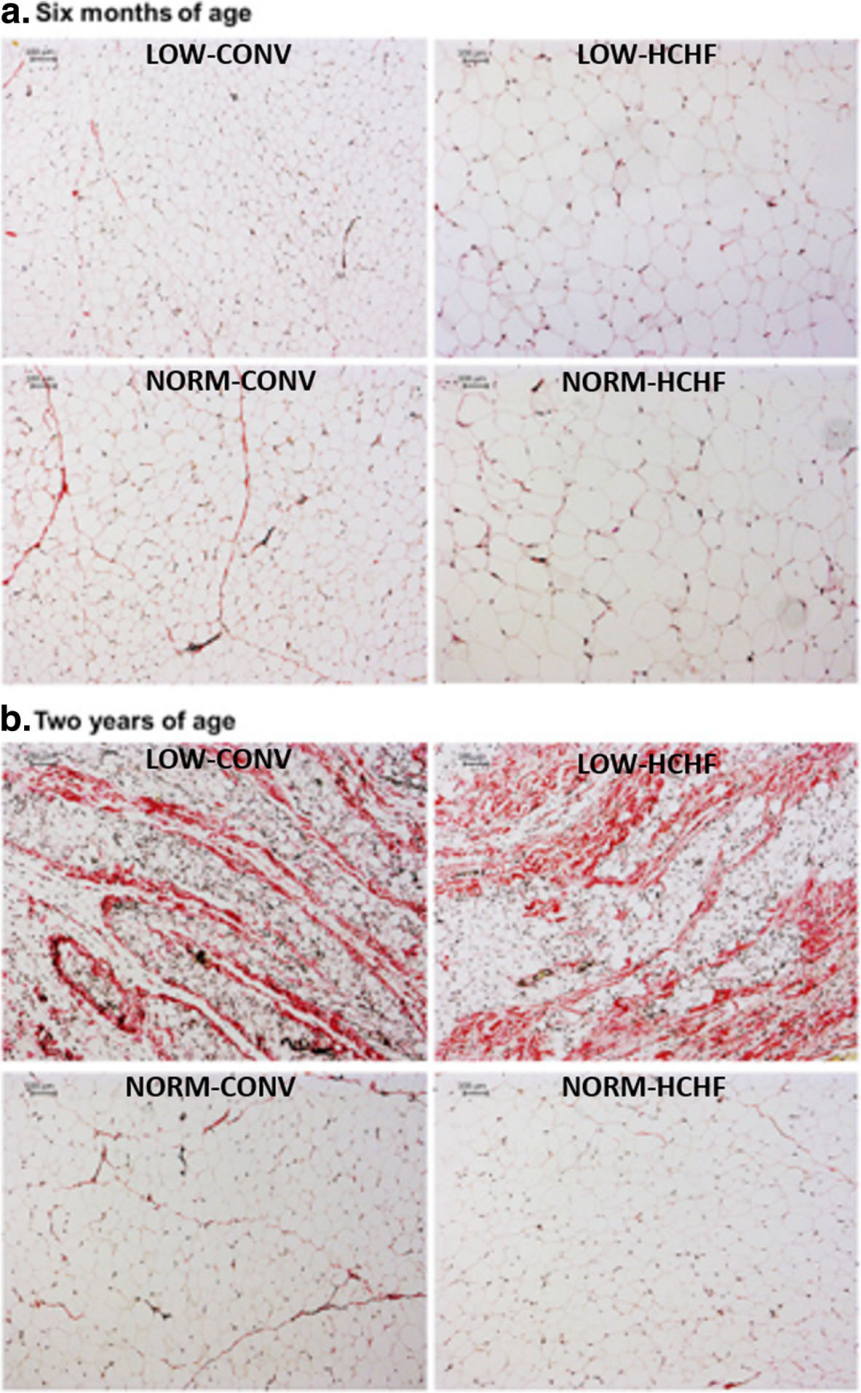
Morphology of Van Gieson-stained subcutaneous adipose tissue from 6 months old adolescent lambs and 2 years old adult sheep (obtained from Nielsen et al. [39]). Panel A: examples of pictures from the 4 groups of lambs, used to grade cell size (and with negligible collagen infiltration) showing a larger population of very small cells in the LOW/CONV group (bottom left) relative to the other groups, and extensive hypertrophy in adipocytes from HCHF lambs (pictures to the right). Panel B: morphological characteristics observed in slides from adult LOW sheep, which was not restricted to a specific early postnatal diet (pictures at the top) with extensive collagen infiltration (grade 4), which was never observed to the same extent among NORM sheep (max grade assigned = 2). For HIGH, NORM, LOW, CONV, HCHF see legends for Fig. 1

**Fig. 4:**
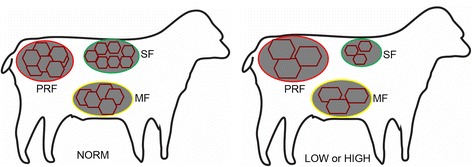
Impacts of late gestational nutrition on hyperplasia and hypertrophy of different adipose tissue depots in adolescent (6 months old) offspring (obtained from Khanal et al.*,* unpublished data). SF, subcutaneous fat (encircled as green); MF, mesenteric fat (encircled as yellow); PRF, perirenal fat (encircled as red). For HIGH, NORM and LOW, see legends for Fig. 1. Each hexagonal structure represents an individual adipocyte

From studies using our Copenhagen sheep model, we have earlier reported that both prenatal overnutrition (150% of energy and 110% of protein requirements) and undernutrition (50% of energy and protein requirements) during late pregnancy led to changes in fat deposition patterns in adolescent offspring (~6 mo of age) resulting in a greater preference for deposition in the abdominal (mesenteric or perirenal) rather than subcutaneous region when the lambs were fed a high-fat diet in early postnatal life [9, 10]. This could be ascribed to a reduced ability to increase fat deposition in subcutaneous adipose tissue during fatness development (Fig. 2). Moreover, in the 6 months old lambs with a history of late gestation undernourishment, an increased occurrence of collagen and non-collagen extracellular matrix, together with greater numbers of a subpopulation of very small adipocytes (<40 μm diameter) was observed in the subcutaneous fat (Fig. 3) [39]. Our recent data also indicate that altered fat distribution patterns due to late-gestation under- as well as overnutrition, followed by exposure to a high-fat diet in early postnatal life are associated with markedly increased perirenal adipocyte hypertrophy (Khanal et al.*,* unpublished data; Fig. 4).

A limited expandability of subcutaneous adipose tissue may give rise to increased intramuscular fat deposition during fattening. This would be consistent with findings in pigs, where low birth weight pigs (1.05 vs. 1.89 kg) had increased lipid deposition (25%) in *semitendinosus muscle* when subcutaneous fat deposition increased, and lean meat content and fibre numbers (19%) were lowered compared to high-birth weight animals [40]. In contrast, other findings in sheep have shown that maternal overnutrition (~50% above maintenance energy requirements) during late pregnancy increased relative subcutaneous fat deposition and leptin expression in subcutaneous and perirenal fat in 1 mo old lambs [7]. The reasons for these apparently conflicting results are not known, but the postnatal diet could have had an influence.

## Impacts of maternal malnutrition on metabolic and endocrine function in growing animals

Malnutrition during gestation has been linked to substantial changes in metabolic and endocrine functions postnatally, and in the following sections implications of prenatal nutritional for glucose-insulin homeostasis, hepatic function and other endocrine functions will be addressed.

### Glucose-insulin homeostasis

The glucose-insulin regulatory axis has long been known to be an important target of foetal programing in humans. In our Copenhagen sheep model we have shown that this is also the case in sheep, and that both under- and overnutrition in late gestation can change the function of this axis permanently. The digestive system and intermediary metabolism of the ruminants differ from the non-ruminant animals, as ruminants ferment most of the dietary carbohydrates to short-chain volatile fatty acids using microbial activity in the rumen leaving only little glucose available for intestinal absorption [41]. However, like in other animal species, hormonal regulatory mechanisms for the maintenance of stable blood glucose level appear to be quite similar in both ruminant and monogastric animals [42, 43]. In our studies, we have performed in vivo metabolic and endocrine tolerance tests to elucidate glucose-insulin-axis function in sheep.

Late gestation undernutrition (50% of energy and protein requirements) decreased peripheral insulin sensitivity in young lambs, but their ability to clear intravenously administered glucose was maintained due to a compensatory upregulation of insulin secretion [44, 45]. However, upon exposure to a high-fat diet in early postnatal life, the ability to clear glucose was reduced, since the high-fat diet interfered with the compensatory upregulation of glucose secretion from the pancreas [45]. The high-fat diet also gave rise to very high plasma levels of triglyceride (~2.0 vs. 0.5 mmol/L) in the lambs, and development of pancreatic fibrosis [45], which to our knowledge has not been reported in ruminant animals previously.

Late gestation overnutrition also affects glucose-insulin regulation, but in a different way. In our studies, lambs exposed to overnutrition in late gestation had increased postnatal gluconeogenic ability in response to intravenous propionate injection [46] and had higher levels of plasma glucose during 44-h fasting when exposed to an early postnatal high-fat diet [10], which was not observed in lambs that had been exposed to undernutrition in late gestation. The underlying mechanims are not known.

### Hepatic function

The liver is an important organ for integration of metabolic pathways, and studies in sheep have shown that prenatal over- and undernutrition may have long-term and differential impacts on hepatic lipid accumulation, glucose and lactate release, and cholesterol synthesis. Nutrient restriction (50% of requirements) during early pregnancy led to increased hepatic lipid accumulation in obese 1 years old sheep offspring [47], and in 4 months old lambs born to dams that were obese around the periconceptional period, expression of genes encoding for factors involved in hepatic fatty acid oxidation was increased [48]. The implications for hepatic function in the offspring later in life were, however, not studied. We ourselves observed increased plasma cholesterol levels in prenatally undernourished 6 months old sheep offspring provided a high-starch-high-fat diet during early postnatal life [46], which may be due to upregulated hepatic cholesterol metabolism. As with glucose metabolism, the impacts of an early postnatal high-starch-high-fat diet in our studies were additive to those of prenatal undernutrition with regard to lipid metabolism and hepatic function. This is because the predisposition of higher plasma cholesterol levels due to late gestation undernutrition (4.3 vs. 1.6 mmol/L) was manifested particularly upon additional exposure to a postnatal high-starch-high-fat diet in adolescent lambs [46]. Our studies have thus shown clear indications that late gestation undernutrition impacts on postnatal cholesterol metabolism in sheep offspring but further investigation into the underlying mechanism and its potential long-term implications for farm animals is warranted in future.

### Other endocrine functions

Not only glucose-insulin axis function, but also a number of other endocrine systems are subject to programming in foetal life, such as the hypothalamic-pituitary-adrenal (HPA) axis [49], the growth hormone (GH)-insulin-like growth factor 1 (IGF-1) axis [50], leptin regulation [51] and the hypothalamic-pituitary-thyroid hormone (TH) axis [52]. We have, however, found that the impacts of prenatal nutrition on basal plasma levels and the adaptive responses to fasting in these endocrine systems became manifested in adulthood only, and were not evident in lambs at 6 mo of age [53]; this will be addressed in the following section.

## Long-term consequences of early life nutrition in adult animals

In animal production systems, animals are kept until adulthood for special production purposes, such as reproduction and lactation [54]. Much less information is available regarding the implications of FP in adulthood in farm animals, probably due to the fact that it is costly to run such studies for several years. Prevailing evidence suggests that many of the implications of FP become increasingly manifested as the animal approaches adulthood. As already mentioned, low birth weight in lambs can be compensated for early postnatal catch-up growth so that normal slaughter weights can be achieved. However, adult body composition can be altered as demonstrated by increased adult adiposity and body weight [55], and there is also, as previously mentioned, evidence suggesting that linear growth can be terminated earlier, resulting in smaller adult body size [27]. In our sheep studies, we did, however, not observe changes in neither body size nor proportions or weights of major organs or muscle or adipose tissue mass in adult offspring that had been exposed to over- or undernutrition in their late foetal life [56], except for increased adrenal weights [9]. Irrespective of that, abnormal subcutaneous morphology (more extensive fibrosis and the occurrence of a subpopulation of very small adipocytes) was also clearly evident in prenatally nutrient-restricted adult offspring regardless of postnatal diet [39]. These morphological changes were similar to the previously mentioned morphological changes observed in non-obese 6 months old lambs. We were to our knowledge the first to report that prenatal undernutrition has long-term implications for composition of fatty acids in skeletal muscle, liver and adipose tissues [35, 39, 57]. Thus, sheep with a history of prenatal undernutrition reduced the myristic acid content and increased the C16:0 to C18:0 fatty acid ratio in perirenal fat, an effect which was not observed in lambs [39]. The underlying reasons for such a FP of fatty acid composition in tissue lipids are unknown, and this could not be ascribed to differences in the postnatal diet. Tissue levels of myristic acid appear to be rate-limiting for a process termed protein myristoylation, and myristoylation impacts function of appr. 0.5–3% of the human proteome [58], and the consequences of such changes thus merit to be addressed in future studies. Prenatal undernutrition was also associated with increased triglyceride, ceramide and free fatty acid contents in livers of adult sheep, which was not observed in lambs [57].

Although studies focusing on early life nutritional impacts in adult offspring are relatively scarce, it seems reasonable to conclude that (subcutaneous) adipose tissue morphology and expandability (increased extracellular matrix, abundance of very small adipocytes) as well as hepatic and adipose lipid composition appear to be a permanent target of FP induced by late gestational undernutrition.

### Glucose-insulin axis function

The functionality of a whole range of endocrine systems is altered in animals subjected to maternal malnutrition, but long-lasting impacts appear to be less pronounced following prenatal overnutrition as compared to undernutrition. Undernutrition during late, but not early, gestation in sheep led to impaired insulin sensitivity of peripheral tissues (reduced glucose tolerance) in adult offspring alongside increased adipose tissue mass [59]. This is consistent with our previous studies, where we found that a depression of insulin sensitivity [45], reduced pancreatic insulin secretory capacity as well as plasticity of down-regulation of insulin secretion [60] persisted into adulthood in sheep with a history of late gestation undernutrition.

In contrast, the adverse impacts of an early postnatal high-fat diet on glucose-insulin axis function, which were clearly observed in lambs (poorer glucose tolerance, reduced insulin secretion and clearance ability), completely disappeared in adult sheep when they were shifted from a high-starch-high-fat to a normal diet and normalization body fat contents [45].

In ruminants as in monogastrics, insulin is the hormone responsible for stimulating transport of glucose into insulin-sensitive tissues, where skeletal muscle and adipose tissues are the most important, but not into insulin-insensitive tissues including tissues important for reproduction (mammary gland and the conceptus). Thus, poor insulin sensitivity and reduced plasticity of pancreatic insulin secretion in sheep exposed to undernutrition in late foetal life, can undoubtedly influence how different tissues are adjusted or prioritized for the glucose utilization during reproductive cycle, pregnancy, etc. [61] and hence the (re) production potential of animals.

### Thyroid-hormone axis function

Studies on prenatal nutritional impacts on TH axis function in farm animals are very scarce, although these hormones affect the adaptation and maintenance of a wide range of body functions under different environmental conditions [62] and also play an important role in ending seasonal reproduction in ewes [63]. In one study, adult hyperthyroidism was observed in adult sheep exposed to late gestation undernutrition and this was associated with increased thyroid expression of genes regulating TH synthesis and deiodination. It also increased the number of TH receptors and deiodinase mRNA expression in different target tissues such as liver, cardiac muscle and *longissimus dorsi* muscle but decreased the number of TH receptors and deiodinase mRNA expression in adipose tissues [64]. This suggests that long-term TH axis function is a target of FP in response to foetal undernutrition during late gestation, but its potential influences on animal production traits remain to be established.

### Other endocrine functions

In our Copenhagen sheep model, alterations in HPA axis function and leptin response were induced by late gestational undernutrition and became manifested in adulthood regardless of the dietary exposures early in postnatal life. Prenatally undernourished male lambs and adult female animals had, as already mentioned, increased adrenal weights [9], and we observed that the adult sheep also had elevated plasma cortisol levels and responded to fasting with a reduction in the cortisol levels [53], in contrast to an expected increase plasma cortisol levels during fasting. This may indicate hyperactivity of the HPA axis, and confirms the previous finding in which increased HPA axis response was observed in adult sheep offspring exposed to a short duration of undernutrition during late foetal life [5]. The GH-IGF-1 axis and adaptations to leptin also appear to be targets of FP. In our prenatally undernourished sheep total plasma IGF-1 concentrations were unexpectedly increased during fasting (presumably due to extended half-life in the blood), whereas plasma leptin concentrations were higher during fasting from much lower levels than in non-programmed sheep [45].

Thus, all the hypothalamic-pituitary axes hitherto studied (TH, GH-IGF-1, HPA) have been shown to be targets of FP. However, the phenotypic manifestation of this programming may not become manifested until the animals approach adulthood, and the consquences for productive functions in adulthood are not well-known. The hypothalamus is a main target for leptin, a hormone produced in white adipose tissues, and hypothalamic binding of leptin can induce changes in all hypothalamic-pituitary endocrine axes in addition to its role in down-regulation of feed intake [65] Considering that FP also induces abnormalities of adipose tissue morphology (fibrosis and very small adipocytes), this has led us to hypothesize that FP may target the entire leptin-hypothalamic-pituitary axis.

It is not known, to what extent overnutrition in late foetal life can predispose for similar long-term impacts on this axis, but it can, as previously reviewed, predispose for increased fat deposition, and the development of leptin resistance, with associated defects in a number of endocrine systems affecting hypothalamic appetite regulators and metabolic function [66].

### Reproductive function

Considering that the nutritional history in foetal life has implications for all aspects of later HPA axes previously studied, it is not surprising that reproductive development during foetal and neonatal life is also affected [67, 68] with consequences for subsequent reproductive function in adulthood. There are, however, relatively few studies on these issues, and it is still not clear to what extent the changes in reproductive function are a consequence of FP targeting reproductive organs directly or there may be indirect effects of altered functions of other endocrine systems and changes in energy metabolism.

It has been shown that both over- (ad libitum feeding) and undernutrition (60% of dietary requirements) during a period of 8 wk prior to oocyte collection in ewes led to reduced oocyte competence and fertilization and poor early embryonic development [69]. Additionally, uundernutrition during the early stages of pregnancy, before and during the period of folliculogenesis, delayed foetal ovarian follicular development in sheep [70]. These findings may explain impaired reproductive funtion in sheep offspring observed in other studies. For example, prenatal undernutrition (50% of energy requirements) during the first 95 d of gestation reduced the ovulation rate in female adult sheep [71]. Furthermore, maternal undernutrition during mid- to late gestation led to a reduction in the number of large corpora lutea in female sheep offspring [72]. Nutrition of ewes during late pregnancy or lactation can also influence subsequent lifetime reproductive performance of the female offspring through impact on the ability to sustain pregnancy, i.e. avoidance of embryo or foetal loss [73].

Although it appears evident that maternal undernutrition, both in the preconceptional period and during gestation, can have adverse effects on the overall reproductive function of the offspring, much remains to be understood about the impacts of prenatal overnutrition and gestational stage-specific influences on the development of reproductive function. Studies are also required to ascertain whether lactation performance may be affected by sub-optimal nutrition during foetal life.

### Epigenetic changes due to maternal nutrition during gestation

Epigenetic regulation of gene expression, i.e. DNA methylation, histone modification etc., could be a potential mechanism linking foetal malnutrition to subsequent phenotypic changes in postnatal life (see reviews [74, 75]). Periconceptional undernutrition in sheep has been shown to induce epigenetic changes, namely histone acetylation and promoter methylation, of foetal hypothalamic genes including glucocorticoid receptors and proopiomelanocortin genes [76], which ultimately affects food intake and energy expenditure after birth. Additionally, periconceptional undernutrition has been associated with epigenetic changes in the adrenal *IGF2/H19* genes coexisting with adrenal overgrowth in offspring [77], which may predispose for postnatal susceptibility to stress. Periconceptional restriction of maternal vitamin B and methionine supply led to altered methylation at CpG islands in the foetal sheep liver and with increased adult body weight and fatness of the offspring [78]. The detailed molecular biological mechanisms underlying epigenetic modifications in response to foetal life malnutrition are still poorly understood. Future studies are needed to identify the impacts of prenatal malnutrition at different gestational stages on tissue-specific epigenetic changes and long-term implications of such epigenetic modifications induced in foetal for animal production and performance.

### Can dietary intervention later in life reverse the adverse programming outcomes of early life nutrition?

An important issue in animal production is to what extent undesirable effects of early life malnutrition can be minimized or completely reversed by dietary or other interventions later in life. Unfortunately, studies investigating the possibility of reversing undesirable FP outcomes with dietary interventions later in life are scarce and not encouraging. We have shown in sheep that it was possible to effectively reverse the adverse outcomes (in terms of increased body fat, higher plasma lipid profiles, poor glucose-insulin homeostasis etc.) induced by an unhealthy fatness-inducing diet fed in early postnatal life if the diet was changed later in life to a normal (for sheep) grass-based diet. Late gestation undernutrition, however, induced permanent programming outcomes [9, 45], particularly on lipid and urea metabolism as described previously, and these implications were more strongly manifested in adult sheep than lambs, irrespective of changes in the postnatal diet.

Extremely few studies have focussed on the long-term impacts of late gestation overnutrition in farm animal species, but it appears from our studies that the possibility of recovery from undesirable nutritional programming outcomes is more likely in individuals exposed to late gestational over rather than undernutrition. The alterations observed in body fat composition and glucose-insulin homeostasis in young lambs with a history of foetal overnutrition did not persist into adulthood [56].

## Conclusion and future perspectives

**Fig. 5:**
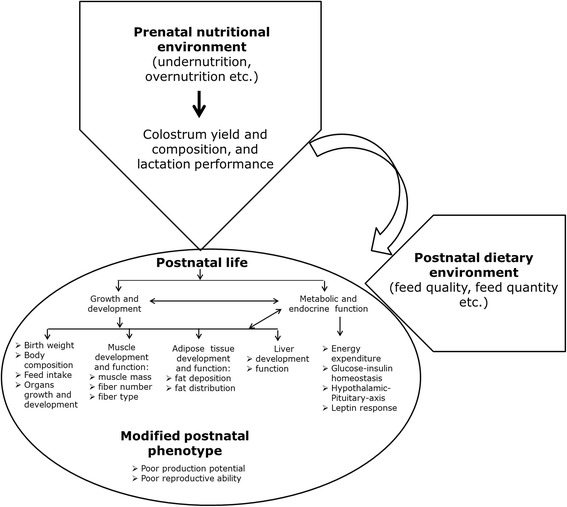
Impacts on early life nutrition on animal physiology and metabolism.

Foetal or developmental programming can have life-long impacts on the health and disease status of farm animals (Table 2), thus affecting the economy of livestock production (Fig. 5). Indeed, it has earlier been reported that foetal programming can be treated as a management tool to improve the livestock productivity in commercial farming but long-term programing impacts specific to different gestational stages and their interactions with postnatal nutritional environment are known [79]. Here, we highlight that foetal programming may be induced during any time point from prior to conception until birth, but the exact manifestation of the foetal programming later in life will depend on the timing of the insult relative to the critical time windows during which embryo formation, placental growth and foetal organogenesis take place. Serious maternal malnutrition during the earlier parts of gestation can influence the development of reproductive functions and muscle fibre numbers and characteristics, but this is probably not very likely to occur under normal production conditions.

**Table 2:** Major impacts of foetal programming due to abnormal nutrition applied at different stages of gestation and under various experimental conditions in sheep

Experimental conditions (gestational age and nutritional environment)	Primary changes in postnatal life	Reference
Growth characteristics		
Late gestational (105 d to term) overnutrition (150% energy and 110% protein) or undernutrition (50% energy and protein) + Early postnatal high-fat diet (0 d to 6 mo)	Reduced birth weight due to prenatal undernutrition, but no impacts due to prenatal overnutrition; Increased abdominal and perirenal fat deposition relative to subcutaneous fat by prenatal under- and overnutrition	[10]
Late gestational (105 d to term) undernutrition (50% energy and protein) + Early postnatal high-fat diet (0 d to 6 mo)	Reduced birth weight; Increased TG, ceramide and free fatty acids in liver, increased extracellular matrix content and very small adipocytes proportion in subcutaneous fat, hyperthyroidism and increased adrenal weights in prenatally undernourished adult sheep (2 yr)	[9, 39, 57, 64]
Late gestational (100 d to term) undernutrition (70% of energy requirements)	Reduced birthweight (18%) and weaning weight, but no weight differences in adulthood (26 wk)	[21]
Late gestational (115 d to term) overnutrition (133% energy)	Increased relative subcutaneous deposition in 1 months old lamb	[7]
Late gestational (109 d to term) undernutrition (50% of energy and protein)	Lowered colostrum yield	[24]
Late gestational (105 d to term) undernutrition (50% of energy and protein)	Lowered birth weight, colostrum and milk yield (lactation performance)	[25]
Mid-gestational (85 d to 115 d) undernutrition (50% of energy requirements)	Decreased muscle weights in newborn lambs	[33]
Early to mid-gestational (28 d to 78 d) undernutrition (50% of requirements)	Increased intramuscular fat content in skeletal muscle in 8 mo old offspring	[15]
Early to mid-gestational (30 d to 70 d) undernutrition (50% of energy requirements)	Fewer fast and more slow muscle fibres in newborn lambs	[33]
Early to mid-gestational (30 d to 80 d) undernutrition (50% of energy requirements) + Postnatal obesogenic environment (restricted physical activity) from weaning (10 wk) to 1 yr	Increased hepatic TG accumulation in prenatally undernourished, obese adult sheep (1 yr.)	[47]
Metabolic and endocrine function		
Late gestational (105 d to term) over- (150% energy and 110% protein) or undernutrition (50% energy and protein) + Early postnatal high-fat diet (0 d to 6 mo)	Reduced glucose clearance and increased glucogeneogensis in matched prenatally overnourished high-fat fed lambs; Increased cholesterol levels in mismatched prenatally undernourished high-fat diet fed lambs and adult sheep	[10, 46, 56]
Late gestational (from 105 d to term) undernutrition + Early postnatal high-fat diet (0 d to 6 mo)	Reduced insulin sensitivity and increased insulin secretory responses to glucose in prenatally undernourished lambs; Poor glucose tolerance in mismatched prenatally undernourished high-fat fed lambs (mismatch group); Poor insulin clearance in prenatally undernourished high-fat fed adult sheep	[45]
Late gestational undernutrition (from 105 d to term)	Reduced insulin secretory ability with increased compensatory insulin sensitivity in 19 wk. old lambs	[44]
Late gestation undernutrition (from 110 d to term)	Poor glucose tolerance in adult sheep (1 yr)	[59]
Late gestational overnutrition (from 115 d to term)	Increased leptin expression in subcutaneous and perirenal fat from 1 months old lamb	[7]
Reproductive function		
Early gestational (0 d to 95 d) undernutrition (50% energy)	Reduced ovulation rate in prenatally undernourished adult female sheep (20 mo)	[71]
Early to mid (0 d to 30 d) or mid to late (31 d to 100 d) gestational undernutrition (50% requirements)	Increased number of small follicles in the ovary (early to mid-gestation undernutrition); reduced large corpora lutea (mid to late gestation undernutrition) in 10 mo old female lambs.	[72]

On the other hand, in animals born precocial, such as sheep, around three quarters of the growth of the foetus [80], and of the mammary gland of the dam [24], occurs during the last 2 mo of gestation. For that reason, adverse FP is much more likely to occur in animal production systems, and with more severe consequences, during late- rather than early- to mid-gestation [8, 81, 82], if adequate attention is not paid to the nutrition of the pregnant dam [83] particularly during multiple pregnancy. Indeed, this may be the case in many parts of the world, where the late gestation period coincides with poor grazing conditions, e.g. during the dry season in tropical countries [84, 85] or the winter in the Nordic and alpine regions [86]. Late gestation malnutrition can have a wide range of both short-term (birth weight, weaning and slaughter weight, and glucose-insulin regulation) and long-term (metabolic and endocrine function, including but not limited to the glucose-pancreatic-hepatic and adipose-hypothalamic-pituitary axis functions, adipose development, fatty acid composition, and reproduction) consequences.

In ruminant production systems, young animals used for meat production are slaughtered within months of birth to obtain the best slaughter result in terms of economic return and meat quality [87]. It can be anticipated that impacts of adverse nutritional programming in utero are of minor quantitative significance at this age, unless the animal has been severely affected during foetal life. However, the timing of abnormal nutrition exposures in utero and the early postnatal nutrition can have implications for lean-to-fat ratios in slaughter animals, since morphogenesis of muscle in precocial species takes place, and may be programmed during the earlier parts of gestation, whilst development of adipose tissue development occurs later in gestation and into early postnatal life [88].

It has earlier been acknowledged that foetal programming in response to severe or prolonged improper nutrition is likely to affect various production traits in commercial sheep farming [79]. Here, we suggest that the best way to manage prenatally programmed animals, particularly undernourished animals (birth weight deviation by 15–20% of the normal range), is to destine them for slaughtering, and not allow them to enter into production processes taking place in adulthood, since the major adverse implications of FP become manifested later in life [45, 89]. Furthermore, there is a risk that undesirable traits may be transferred to future generations due to epigenetic inheritance, and it is therefore advisable to apply proper strategies to avoid the entry of adversely programmed animals into reproduction [90, 91].

Our recent findings suggest that a moderate diet and lower body fat content later in life can prevent or reverse a large part of the impacts induced by fatness development in early postnatal life [56]. FP due to late gestation undernutrition, however, has irreversible life-time impacts on offspring, which are exacerbated upon transient fatness development in early postnatal life. Further studies are needed to confirm the findings from our studies that late gestational overnutrition has fewer long-term detrimental consequences for animal production than foetal undernutrition.

There are unfortunately no biomarkers other than birth weight, which can be used to reliably identify animals (at an early age) that have undergone FP. Although more studies are needed to assess the long-term quantitative impacts and economic consequences of FP, and to find biomarkers and potential means for reversal of such programming outcomes, commercial animal production should now acknowledge this phenomenon and devise management strategies to ensure its prevention and spread to future generations.

## Abbreviations

CONV: Conventional diet
FP: Foetal programming
GH: Growth hormone
HCHF: High-starch-high-fat diet
HIGH: Overnutrition
HPA: Hypothalamic-pituitary-adrenal
IGF-1: Insulin-like growth factor 1
LOW: Undernutrition
NORM: Normal (adequate) nutrition
TG: Triglycerides
TH: Thyroid hormones
